# Chronic obstructive pulmonary disease: Is serum magnesium level a risk factor for its acute exacerbation?

**DOI:** 10.22088/cjim.12.2.223

**Published:** 2021-03

**Authors:** Ketan Kshirsagar, Virendra C Patil

**Affiliations:** 1 Department of Medicine, Krishna Institute of Medical Sciences, Karad , India

**Keywords:** Serum magnesium, Pulmonary diseases, Hypomagnesemia

## Abstract

**Background:**

Determination of modifiable risk factors for treatment and prevention of acute chronic obstructive pulmonary disease (COPD) exacerbation is very crucial. Therefore, the present study determined the role of serum magnesium level in acute COPD exacerbation.

**Methods:**

This prospective study involved 100 patients with an exacerbation of COPD. Demographic data were collected for all the patients, and serum magnesium (Mg^2+^) levels were determined at two different time intervals. These patients were given standard treatment for COPD. All the patients were followed up after one month and later at three-month intervals for one year.

**Results:**

Majority (72%) of the patients had serum Mg^2+^<1.7 mg/dL and the odds of hypomagnesemia was 6.54 times more during exacerbations than when they had stable COPD during follow-up. Patients with serum Mg^2+^<1.7 mg/dL had 9.34 times higher risk of an increased number of acute exacerbations. A significant difference (p<0.05) was observed in the mean serum Mg^2+^ levels and number of COPD exacerbations among patients with hypomagnesemia at different stages of COPD.

**Conclusion:**

Low serum magnesium level during acute exacerbation is correlated with the increased frequency of acute exacerbation in COPD.

Chronic obstructive pulmonary disease (COPD) is a life-threatening lung disease and the third leading cause of death globally (1). According to the Global Burden of Disease Study, there are more than 251 million cases of COPD (2). More than 90% of deaths due to COPD occur in low- and middle-income countries (2). In India, COPD is the second major cause of deaths due to non-communicable diseases (3). The worldwide increase in prevalence of COPD makes its exacerbation an increasingly important phenomenon for patients along with clinicians. As a result, there is a mounting interest not only in designing optimal COPD treatment approaches but also in preventing its exacerbations (4). Magnesium (Mg^2+^) is an intracellular cation that regulates the bronchial tone and respiratory muscle function. Consequently, Mg^2+^ deficiency can lead to exacerbations of pulmonary diseases as it helps in alleviating bronchospasm (5). The relationship between serum Mg^2+^ levels and worsening of COPD symptoms has not been explored thoroughly (6). Moreover, only a limited number of studies on the effect of Mg^2+ ^on the frequency of acute COPD exacerbation have been reported (7). Hence, this study was undertaken to determine the role of serum magnesium level in acute COPD exacerbation.

## Methods

After obtaining approval from the institutional ethics committee (Approval number: KIMSDU/IEC-307/026/06/11/2012), this prospective study was conducted at a private medical college in Karad (Maharashtra) over a period of one and a half year (January 2013-June 2014). Informed consent was obtained from all the patients in the study. 

Hundred patients of either gender, aged ≥30 years, presenting with an exacerbation of COPD, and requiring hospitalization were included in the study. Patients with associated conditions such as gastrointestinal disease, peptic ulcer disease, pancreatitis, pregnancy and lactation; hormonal diseases; renal failure; use of drugs such as thiazide diuretics, loop diuretics; any malignancy; alcoholism etc.; pulmonary embolism, acute exacerbation of bronchiectasis, etc., were excluded from the study. The patients were diagnosed according to the European Respiratory Society Task Force Recommendations (8) for COPD based on the results of dynamic pulmonary function test, i.e., ratio of 1-second forced expiratory volume/forced vital capacity (FEV1/FVC<70). Furthermore, the patients were diagnosed with acute exacerbation based on symptoms, i.e., either severe cough with or without increased volume of sputum or presence of shortness of breath (9, 10). Standard treatment according to GOLD guidelines (Global Strategy for the Diagnosis, Management and Prevention of COPD) (11) was given to the patients. 

Data regarding age, gender, smoking history, medical history, and staging of COPD (GOLD criterion method adopted for staging [stage 1, 2, 3, 4]) (11) were collected for all the patients. Blood samples were collected at two different time intervals for determining Mg^2+^ levels: first when the patient was hospitalized with an exacerbation, and the second when the patient attended the outpatient department with stable COPD during follow-up.

The serum magnesium levels were analyzed by the Calmagite spectrophotometric technique (12). In our laboratory, the reference range for serum Mg2+ level was 1.7 mg/dL to 2.4 mg/dL. A serum Mg2^+^ level of less than 1.70 mg/dL was considered as hypomagnesemia. Statistical analysis was performed by using R software (Version. 3.6.0). Data was expressed as mean and standard deviation, frequency, and percentage. Chi-square test of independence and unpaired *t*-test were used. Correlation between variables was performed by Pearson’s correlation coefficient (r). Data was considered statistically significant when *P*≤0.05.

## Results

 Out of 100 patients included in the study, majority were 61-70 years with males being predominant (male: female=2.8:1). Most of the patients with COPD were smokers. While staging the condition of COPD, it was noted that most of the patients were at stage 2, i.e., moderate COPD (table 1). 

**Table 1:** Distribution of age, sex, smoking history, COPD staging

**Demographics**	**Frequency (%)** **(N=100)**
**Age distribution (years)**	
≤50	6 (6)
51- 60	21 (21)
61-70	45 (45)
71-80	26 (26)
> 80	2 (2)
**Gender**	
Female	26 (26)
Male	74 (74)
**Smoking habits**	
Non-smoker	29 (29)
Smoker	71 (71)
**Stages of COPD (**11)	
STAGE 1 (Early)	1 (1)
STAGE 2 (Moderate)	45 (45)
STAGE 3 (Severe)	40 (40)
STAGE 4 (Very severe)	14 (14)

COPD-Chronic obstructive pulmonary diseases; %-Percentage

**Table 2:** Occurrence of hypomagnesemia in patients

**Serum magnesium levels (mg/dL) **	**Hypomagnesemia**	**f (%)** **(N=100)**
**During COPD condition**		
< 1.7	Yes	72 (72)
≥ 1.7	No	28 (28)
**After recovery (during follow-up)**		
< 1.7	Yes	1 (1)
≥ 1.7	No	99 (99)

f-Frequency; %-Percentage; mg/dL-Milligrams per Decilitre

Most patients had serum Mg^+2 ^levels of <1.7 mg/dL, indicating hypomagnesemia. However, almost all patients during follow-up had serum Mg^+2 ^levels >1.7 mg/dL indicating recovery from hypomagnesemia (table 2). No significant correlation was observed between the occurrence of hypomagnesemia and normomagnesemia, and parameters like age, gender, and smoking history (*p*>0.05) (table 3). A difference was observed in the number of acute exacerbations among the patients as those patients with serum Mg^2+^<1.7 mg/dL had greater number of acute exacerbations (96%) as compared to patients with serum Mg^2+^>1.7 mg/dL (4%). Additionally, the risk ratio (RR) interpretation demonstrated that patients with serum Mg^2+^<1.7 mg/dL had 9.34 times higher risk of having an increased number of acute exacerbations. No significant correlation between serum Mg^2+^<1.7 mg/dL and FEV1/FVC ratio was observed (r =0.52; *P*=0.52). A significant difference was observed in the mean serum Mg^2+^ levels among patients with hypomagnesemia and normomagnesemia, respectively, at different stages of COPD (p<0.05). This indicates that with each increasing stage of COPD, the levels of serum Mg^2+^ were further decreasing in hypomagnesemia as well as normomagnesemia. However, in normomagnesemia, the levels of serum Mg^2+^ were always in the normal range (i.e.,>1.7 mg/dL) (table 4).

**Table 3:** Correlation between baseline parameters and occurrence of hypomagnesemia and normomagnesemia in patients

**Baseline parameters **	**Hypomagnesemia** **(n=72)**	**Normomagnesemia ** **(n=28)**	**P value ** ^c^
**Age (years) (Mean±SD)**	66.54±8.32	66.18±8.0	0.84
**Gender (%)**			
Male	55 (76.38)	19 (67.85)	0.53
Female	17 (23.62)	9 (32.15)	
**Smoking habit (%)**			
Smoker	53 (73.61)	18 (64.28)	0.49
Non-smoker	19 (26.39)	10 (35.72)	

C: Chi-square test; SD: Standard deviation; %: Percentage

**Table 4:** Comparison of COPD stages with mean serum magnesium levels in hypomagnesemia and normomagnesemia cases

**Stages of COPD **	** Hypomagnesemia** **(N=72) **		**Normomagnesemia** **(N=28) **		**P value** ^*^
	**n (%)**	**Serum Mg** ^2+^ ** levels** **(Mean±SD)**	**n (%)**	**Serum Mg** ^2+^ ** levels ** **(Mean±SD)**	
Stage I	0	0	1 (3.6)	1.8±0	-
Stage II	25 (34.7)	1.484±0.14	20 (71.4)	2.03±0.29	<0.001
Stage III	35 (48.6)	1.41±0.12	5 (17.9)	1.82±0.11	<0.001
Stage IV	12 (16.7)	1.33±0.11	2 (7.1)	1.9±0.14	0.04
Total	72		28		

N-number; %-percentage, COPD-Chronic obstructive pulmonary diseases; *- Independent t-test; SD: Standard deviation

## Discussion

The primary aim of the present study was to determine the role of serum magnesium level in acute COPD exacerbation. Most patients were in the age group of 61-70 years with male predominance. This is in accordance with the study conducted by Singh et al. (10). It could be attributed to smoking.

As most patients were smokers, smoking is one of the risk factors. Similar findings were observed by Kanimozhi and Sujatha (13). Smoking causes an accelerated decline of FEV1. Consequently, in patients with early COPD, the cessation of smoking can improve lung function and slow down the annual decline of FEV1 (14). Hypomagnesemia is encountered frequently in patients developing acute exacerbation. In this study, most of the patients had hypomagnesemia due to low levels of serum Mg^2+^. It is in line with findings observed by Pham et al. (15). Stages 2 and 3 were the most common stages of COPD, indicating that most of these cases were either at the moderate or severe stage. Other studies have also shown similar results (12). Stages 2 and 3 of COPD are usually associated with hypoxemia, which when combined with chronic respiratory insufficiency, can cause hypomagnesemia and magnesium depletion (8). Further, among all the study cases diagnosed with acute exacerbated COPD, most of the patients with hypomagnesemia were having stage 2 and stage 3 disease and few were in stage 4. Similar findings were observed in other studies where majority of hypomagnesemia patients were in stages 2 and 3 (12, 16). A negative correlation was noted between FEV1 and the number of exacerbations in this study demonstrating that the increase in frequency of exacerbations was decreasing the forced expiratory volume. Also, the patients with serum Mg^2+^<1.7 mg/dL had greater number of acute exacerbations as compared to patients with serum Mg^2+^>1.7 mg/dL. Frequent exacerbations are usually associated with low FEV1 (17). Coa et al.in their prospective study observed that an FEV1 <50% was associated with an increased number of acute exacerbations (18). The increased number of exacerbations noticed in patients with serum Mg^2+^<1.7 mg/dL could be probably due to raised systemic airway inflammation and another possible reason could be due to increased sputum eosinophils in response to bacterial and viral infection (19). Much of the impetus for serum Mg^2+^ recognition, both as a potential therapeutic agent and risk factor in patients with COPD, comes from the fact that Mg^2+^ has a well-established role in alleviating bronchospasm, one of the symptoms of COPD. Mg^2+^ causes relaxation of bronchial smooth muscles (4). In this study, with each advancing stage of COPD, the levels of serum Mg^2+^ were further decreased in hypomagnesemia cases (acute exacerbation of COPD) stipulating an increase in the severity of the disease. The rate of frequent exacerbations also increased. Patients with low serum Mg^2+^ levels had a greater number of acute exacerbations during the advanced stages. This was a significant finding. Other studies also showed similar results (6, 7). Patients with COPD seem to have a decreased bioavailability of serum Mg^2+^. The probable cause of low serum magnesium level in COPD patients might be due to heavy smoking habit, reduced dietary Mg intake or due to the use of drugs that could increase Mg^2+^ deprivation (e.g. cortisones and beta-agonists) (19).

This study has its own limitations. For acute exacerbation of COPD, frequent hospital readmissions and their associated factors were not considered. Further multicentric, studies with a larger sample size and longer follow-up period are required to validate the results. In conclusion hypomagnesemia is a common finding in acute exacerbation of COPD due to the presence of low levels of serum Mg^2+^. Patients with acute exacerbation of COPD usually have advanced disease stage, i.e., stages 2 and 3. We consider the observed association between exacerbation of COPD and serum Mg^+2^ to be substantial. 

## Funding:

This research received no specific grant from any funding agency in the public, commercial, or any profit sectors.
